
JOURNAL INFORMATION
==============================
NLM Title Abbreviation: ScientificWorldJournal
Journal ID: ScientificWorldJournal
NLM Journal ID: 101131163
Journal ID: TSWJ

The Scientific World Journal

ISSN: 2356-6140
EISSN: 1537-744X
Publisher: Wiley

ARTICLE INFORMATION
==============================
PMCID: PMC6084146
DOI: 10.1100/tsw.2001.424
Article ID: 916727
Article version: 1
Subjects: Extended Abstract

Enhanced Vasodilator Responses to CGRP (Calcitonin Gene-Related Peptide) in Subcutaneous Arteries in Human Hypertension

Lind H.
Edvinsson L.
Division of Experimental Vascular Research, Department of Internal Medicin, Lund University Hospital, Lund, Sweden
Publication date: 2001
Electronic publication date: 2001 Aug 17
Volume: 1
First page: 35
Last page: 35
Copyright: Copyright © 2001 H. Lind and L. Edvinsson.
Copyright year: 2001
License: This is an open access article distributed under the Creative Commons Attribution License, which permits unrestricted use, distribution, and reproduction in any medium, provided the original work is properly cited.
License URL: https://creativecommons.org/licenses/by/3.0/

==============================
4th International Meeting on Calcitonin Gene-Related Peptide (CGRP)
*TheScientificWorld* (2001) 1(S1), 35
ISSN 1532-2246; DOI 10.1100/tsw.2001.424

### ENHANCED VASODILATOR RESPONSES TO CGRP (CALCITONIN GENE-RELATED PEPTIDE) IN SUBCUTANEOUS ARTERIES IN HUMAN HYPERTENSION

H. Lind and L. Edvinsson
Division of Experimental Vascular Research, Department of Internal Medicin, Lund University Hospital, Lund, Sweden

### INTRODUCTION

To evaluate alterations induced by essential and renovascular hypertension on vasomotoresponse to CGRP, substance P, potassium and noradrenaline human subcutaneous vessels were studied. Isolated segments (1 to 2 mm) of small subcutaneous arteries (diameter 0.1 to 0.9 mm) and veins (0.1 to 1.0 mm) with intact endothelium from patients (essential n = 10, renovascular n = 6) and controls (n = 17) were used.

### METHODS

In accordance with our previously published method description[1] cylindrical segments (1 to 2 mm long) were mounted on two L-shaped metal prongs, one of which was connected to a force displacement transducer (FT03C) attached to a PowerLab unit for continuous recording of the isometric tension on a PC computer, and the other to a displacement device.

### RESULTS

Enhanced dilatory responses (Emax) but no change in sensitivity ($pEC_{50}$) were demonstrated in arteries but not in the veins to CGRP in hypertensives ($p < 0.01$) as compared to normotensives, and in the hypertensives subgroups (essential hypertension $p < 0.05$; renovascular hypertension $p < 0.05$). The maximum dilatory response in the arteries of the controls was (Emax ± s.e.m.) 48.6 ± 8.0% (see Figure). Corresponding values for hypertensives was 78.7 ± 5.4%, (subgroups; essential hypertensives 77.2 ± 7.2% and renovascular hypertension 82.0 ± 7.6%). The relaxant responses to substance P were not altered neither in arteries nor veins of the hypertensives. Furthermore, there were no differences in the contractile responses to 60 mM potassium or 10 µM noradrenaline between the groups.

### CONCLUSION

The results suggests that the enhanced vasodilator response to CGRP in hypertension is an adaptive reaction. The elevated blood pressure may be augmented by vasodilatory activity since different subgroups of hypertensives showed the same results.

### REFERENCE

1. Högestedt, E.D., Andersson, K.E., and Edvinsson, L. (1983) Mechanical properties of rat cerebral arteries as studied by a sensitive device for recording of mechanical activity in isolated small blood vessels. Acta Physiol. Scand. 117, 49–61.
